# Propranolol Is Associated with Lower Risk of Incidence of Hepatocellular Carcinoma in Patients with Alcoholic Cirrhosis: A Tertiary-Center Study and Indirect Comparison with Meta-Analysis

**DOI:** 10.1155/2020/1892584

**Published:** 2020-04-09

**Authors:** Tzu-Hao Li, Yu-Lien Tsai, Chien-Fu Hsu, Chih-Wei Liu, Chia-Chang Huang, Ying-Ying Yang, Hung-Cheng Tsai, Shiang-Fen Huang, Yun-Cheng Hsieh, Hsuan-Miao Liu, Tzung-Yan Lee, Ming-Chih Hou, Chang-Youh Tsai, Han-Chieh Lin

**Affiliations:** ^1^Division of Allergy, Immunology, And Rheumatology, Department of Internal Medicine, Shin Kong Wu Ho-Su Memorial Hospital, No. 95, Wen Chang Rd., Shihlin District, Taipei 111, Taiwan; ^2^Institute of Clinical Medicine, National Yang-Ming University, No. 155, Sec. 2, Linong St., Beitou District, Taipei City 112, Taiwan; ^3^Faculty of Medicine, National Yang-Ming University, No. 155, Sec. 2, Linong Street, Beitou District, Taipei City 112, Taiwan; ^4^Department of Medicine, Taipei Veterans General Hospital, No. 201, Sec. 2, Shipai Rd., Beitou District, Taipei City 112, Taiwan; ^5^Division of Allergy, Immunology, And Rheumatology, Department of Medicine, Taipei Veterans General Hospital, No. 201, Sec. 2, Shipai Rd., Beitou District, Taipei City 112, Taiwan; ^6^Division of Clinical Skills Training, Department of Medical Education, Taipei Veterans General Hospital, No. 201, Sec. 2, Shipai Rd., Beitou District, Taipei City 112, Taiwan; ^7^Division of Gastroenterology, Department of Medicine, Taipei Veterans General Hospital, No. 201, Sec. 2, Shipai Rd., Beitou District, Taipei City 112, Taiwan; ^8^Graduate Institute of Traditional Chinese Medicine, Chang Gung University, No. 259, Wenhua 1st Rd., Guishan Dist., Taoyuan City 333, Taiwan

## Abstract

Alcoholic cirrhosis (AC) leads to enormous disease burden and occupies a substantial proportion in the etiology of hepatocellular carcinoma (HCC), but scarce attention has been paid to this topic. Besides, propranolol has been reported to decrease the rate of HCC in viral hepatitis. We conducted a retrospective tertiary-center cohort study to identify the HCC incidence in AC patients with or without propranolol. A total of 1,046 AC patients with hospitalization had been screened, and those with regular follow-up for three years or otherwise until the date of malignancy diagnosis without meeting exclusion criteria were enrolled; finally, 23 AC patients with propranolol and 46 AC patients without propranolol were analyzed after twofold propensity-score matching. The cumulative incidence of HCC was lower in the propranolol group (log-rank test, *P* = 0.046). Furthermore, we undertook the meta-analysis of annual incidence of HCC in AC patients, and 1,949 publications were screened, within which eight studies were analyzed; the pooled annual incidence was 2.41%, which was higher than the calculated annual incidence of HCC in our AC cohort with propranolol (1.45%). In conclusion, propranolol is associated with decreased risk of HCC incidence in patients with AC.

## 1. Introduction

Cirrhosis is characterized by various complications, including high risk of developing hepatocellular carcinoma (HCC), and the necessity of policies regarding HCC surveillance has been proposed [1]. According to the cost-effectiveness benefit, an incidence of HCC of at least 1.5% per year should undergo HCC screening [2]. Although a major epidemiological study estimated that approximately 30% of new primary liver cancer cases could be ascribed to alcohol, the annual incidence of HCC in alcoholic cirrhosis (AC) had been reported as a wide range from 0.4 to 5.6% [3–5]; therefore, a more comprehensive investigation should be performed.

Owing to the high risk of HCC in AC, seeking effective medication for HCC prevention is essential. A nonselective beta blocker (NSBB), propranolol, has been proven to have pleiotropic benefits for patients with cirrhosis, including being able to correct portal hypertension, prevent variceal bleeding, reduce the incidence of ascites, and decrease the risk of cirrhotic decompensation; therefore, propranolol is widely used in cirrhotic patients [6, 7]. On the other hand, in the case other than HCC, propranolol possesses antitumor entities in several types of tumor [8–11]; however, there has been scarce literature regarding the influences of propranolol on HCC incidence in AC patients.

As was pointed out above, here in the present study, we undertook a retrospective propensity-score matching (PSM) analysis to investigate whether long-term propranolol treatment had protective effects on the incidence of HCC; furthermore, we conducted a meta-analysis to retrieve the pooled annual incidence of HCC in AC patients for integrative recognition, and comparison of the effects of long-term propranolol use.

## 2. Methods

### 2.1. Subjects

We conducted a retrospective tertiary-center cohort study, of which the protocol was approved by the Institutional Review Board of Taipei Veterans General Hospital. The medical and hospitalization records of AC subjects older than 18 years within the years 2006 and 2017 were reviewed. For the propranolol group, we enrolled subjects who had been hospitalized due to AC and regularly treated with propranolol for three consecutive years or otherwise until the date of HCC diagnosis; for the control (nonpropranolol) group, we analyzed the AC patients who regularly follow up without propranolol treatment. The criteria of exclusion consisted of patients younger than 18, treatment with hepatobiliary surgery or liver transplantation, history of primary liver cancer (HCC or cholangiocarcinoma) or malignancies before enrolment, and the initiation of propranolol use before the year 2006. The follow-up duration of each subject was three years or otherwise until the date of HCC diagnosis according to the available medical records, and those with new-onset malignancy other than HCC or loss of follow-up were censored.

The basic demographic data, including the age, sex, superimposed chronic liver disease, date of cirrhosis diagnosis, initiation date of propranolol, and Child-Pugh scores, were acquired. Comorbidities including hypertension, hyperlipidemia, ischemic heart disease, chronic obstructive pulmonary disease, diabetes mellitus (DM), tuberculosis, and end-stage renal disease were also recorded.

### 2.2. Outcome Measurement

The primary outcome in our study was the incidence of HCC, which was defined according to diagnostic image presentation or tissue proof, along with the corresponding catastrophic illness certificate. As to the catastrophic illness certificate of national health insurance, the medical records and diagnoses were reviewed by independent subspecialists to ensure the accuracy of diagnosis. The diagnosis of malignancy other than HCC and mortality was defined as a secondary endpoint.

### 2.3. Literature Review and Meta-Analysis

In comparison with the previous literature, we identified the relevant studies written in English or Chinese, focusing on the incidence of HCC in AC, from the PubMed, EMBASE, Airiti Library, and Cochrane Central Register of Controlled Trials (CENTRAL) databases, which had been published until April 2019, and conducted a meta-analysis for pooled incidence. The integrative search was undertaken in accordance with the Preferred Reporting Items for Systematic Reviews and Meta-Analyses (PRISMA) guidelines, by means of the term “alcoholic cirrhosis” AND “hepatoma” AND “incidence”; in the Airiti Library, the corresponding Chinese terms were applied for searching. The articles compatible with the inclusion criteria were retrieved, and literature published in English or Chinese was enrolled. Case reports and series, letters to the editor, *in vitro* studies, and studies on experimental models were excluded.

Three independent authors (T.H. Li, Y.L. Tsai, C.F. Hsu, and Y.Y. Yang) screened the abstracts and reviewed the full text adhering to the preset protocols; for disparities in literature appraisal, other authors discussed to achieve consensus. We extracted the author or study name, time of publication, country, number of participants, and incidence of HCC; for the data presented as cumulative incidence, the annual incidence rate was calculated by the formula as previously mentioned [12]. The potential publication bias was assessed by fail-safe N, which presumed the estimated number of studies required to reduce the effect size to a nonsignificant level.

### 2.4. Statistical Analysis

The incidence rate of primary HCC among the study subjects was calculated. In the part of direct comparison between the groups with or without propranolol, we conducted a 1 : 2 pair-matched case-control cohort by means of nearest-neighbor PSM, which was adjusted by age, sex, Child-Pugh score, cirrhosis duration, and comorbidity after estimating the probability by logistic regression. Student's *t*-test and a chi-squared test were applied for continuous and categorical variables, respectively. The Kaplan-Meier method with log-rank test was utilized for comparison of the risks.

The difference of annual incidence between our study and the pooled incidence being deduced from previous literature was assessed using a *P* value by the exact binomial test; *P* values adhered to two-sided tests, and those <0.05 were considered significant. With regard to the meta-analysis of annual incidence, the effect size was derived with a random-effects model. The heterogeneity between studies was examined via Cochran's *Q* test, in which *P* < 0.05 suggested significant heterogeneity. The *I*-square index was utilized for quantification of heterogeneity. Data analyses were performed by means of Statistical Product and Service Solutions V.26 (SPSS, IBM, Armonk, New York, USA) and Comprehensive Meta-Analysis V.3 (Biostat, Englewood, New Jersey, USA).

## 3. Results

### 3.1. Patient Characteristics and Annual Incidence Rate

For the propranolol group, a total of 369 hospitalized patients who had been admitted with the diagnosis of AC and a concurrent prescription of propranolol were identified, and we ruled out those who met the exclusion criteria: 157 subjects with irregular use or discontinuation of propranolol, 77 subjects with insufficient medical records, 29 with previous HCC history, 18 with a history of liver transplantation or hepatobiliary surgery, 17 with propranolol use before year 2006, 11 with malignancy other than HCC, five with misdiagnosis of AC, and one with documented intolerance of propranolol; among the remaining 54 subjects, we included those with newly diagnosed HCC more than six months after enrolment or with regular follow-up for three years; consequently, 23 subjects were enrolled.

For the nonpropranolol group, 677 AC patients with admission were screened; after exclusion by the aforementioned criteria, a 1 : 2 pair-matched PSM was embarked on and yielded 46 subjects. The demographic data of the enrolled subjects is shown in Table 1, exhibiting no significant difference of the baseline variables between the two groups after PSM.

**Figure 1:**
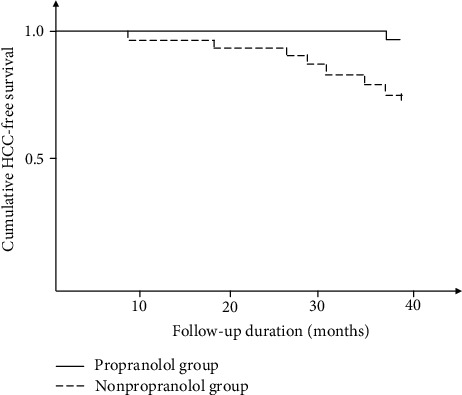
Kaplan-Meier curves for hepatocellular carcinoma-free survival in the propranolol group versus the nonpropranolol control group.

In our three-year observation, one male patient in the propranolol group was diagnosed as HCC in the 35^th^ month; comparatively, eleven patients in the nonpropranolol group were diagnosed as HCC during the 8^th^ to 36^th^ month, and the proportion of HCC incidence was significantly higher than that in the propranolol group (*P* = 0.043). In addition, the Kaplan-Meier analysis also disclosed a significantly higher cumulative incidence of HCC in the nonpropranolol group (*P* = 0.046, Figure 1). Neither incidence of other malignancy nor mortality was found during the follow-up period.

### 3.2. Comparison to the Pooled Annual Incidence Rate of HCC in AC

**Figure 2:**
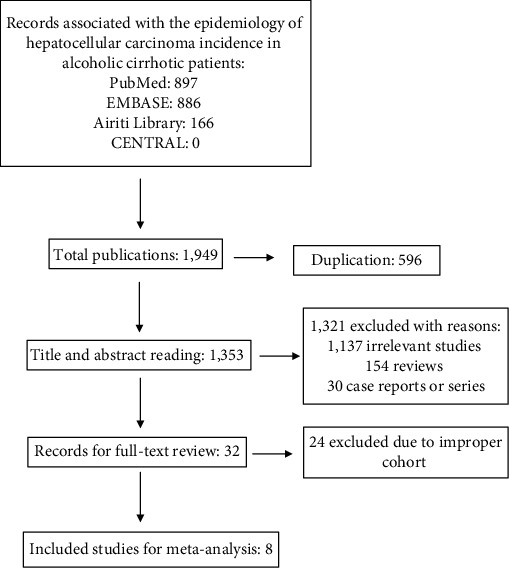
Flow chart of study inclusion for meta-analysis.

A total of 1,949 publications were identified from PubMed, EMBASE, CENTRAL, and Airiti Library, with 596 duplications between these databases. After title and abstract reading, 1,137 citations were considered as irrelevant studies, 154 citations were review articles, and 30 citations were case reports or series. Thirty-two articles were reviewed by full text after the exclusion, and finally, eight studies were included in the present meta-analysis [4, 5, 13–18]. The flow chart is listed in Figure 2.

**Figure 3:**
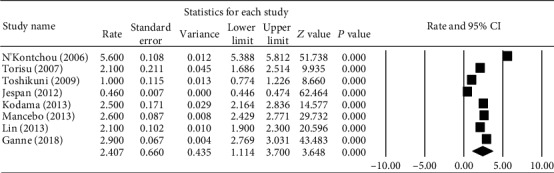
Forest plot of annual incidence rate of hepatocellular carcinoma in patients with alcoholic cirrhosis. The numbers shown in the picture were the percentage of annual incidence.

The eight studies covering 10,361 AC patients contained five European and three Asian works, and the reported annual incidence ranged from 0.4 to 5.6%. The pooled annual incidence in the meta-analysis was 2.41% with high heterogeneity (*I*^2^ = 99.8%, *P* < 0.001, Figure 3). No further metaregression was performed because of the relatively few studies [19]. Publication bias was examined by means of Rosenthal's fail-safe number, which was 5,135, indicating low publication bias.

The annual incidence in our present propranolol group was 1.45%, which was significantly lower than the general pooled annual incidence garnered from the meta-analysis (1.45% vs. 2.41%, respectively, *P* = 0.04). Namely, propranolol treatment in AC patients demonstrated reduction in the general annual incidence rate of HCC during the three-year follow-up.

## 4. Discussion

In the present study, propranolol treatment was associated with lower incidence of HCC in the AC group compared to the nonpropranolol group; moreover, we performed the meta-analysis of HCC incidence in AC patients to obtain more explicit data and further examined the effects of long-term propranolol use on HCC incidence. Herein, significant reduction of HCC incidence was embodied in our research.

Beta-adrenergic signaling plays a role in tumorigenesis and angiogenesis, and propranolol has manifested antitumor effects in various tumors [8–11, 20, 21]; as for *in vitro* studies of HCC, propranolol showed inhibition of proliferation and promotion of apoptosis in hepatoma cell lines [22]. However, data concerning the effects of propranolol on HCC incidence in cirrhotic patients obtained from clinical observational research remained controversial; some studies reported that the likelihood of developing HCC was lower in propranolol-treated patients than in those not treated, but other works indicated no significant difference in HCC incidence and survival [23, 24]. Recently, one work aimed at the patients with cirrhosis on the waiting list for liver transplant yielded a protective effect for HCC, among whom 18 patients were attributed to alcohol abuse; however, these alcoholic subjects were not analyzed individually [25]. Actually, the aforementioned studies enrolled cirrhotic patients regardless of etiologies and did not conduct subgroup analysis according to etiology, thus lacking data specific to AC patients.

Our work suggested that propranolol lowered the incidence of HCC in AC, of which the pathogeneses were mainly on the hepatic alcohol-mediated carcinogenesis resulting from the detrimental entities of acetaldehyde and the dysregulation of methylation, as well as immune alteration resulting from chronic inflammation [26]; these mechanisms were distinct from other etiologies such as viral hepatitis, and the antitumor effects might differ. In previous literature, propranolol had been mentioned with regard to counteractive properties to acetaldehyde [27, 28], modification of aberrant methylation pertinent to tumorigenesis [29], and anti-inflammatory roles in cirrhotic circumstances [30]; thus, the preventive effects on the HCC incidence of propranolol were theoretically grounded.

Despite the substantial proportion and enormous disease burden of AC, less research attention has been paid to it than to other etiologies such as viral hepatitis and nonalcoholic fatty liver disease [31]. Except for the well-known prognostic benefits of NSBB including propranolol for cirrhotic patients with portal hypertension, in patients with AC and alcoholic cardiomyopathy or congestive heart failure, NSBB improves quality of life, hemodynamics, and ejection fraction and reduces the severity of pulmonary hypertension [32, 33]. In addition, NSBB has also been reported to prevent hepatosteatosis and attenuate progression in animal models of alcoholic liver diseases (ALDs) [34]. In combination with the antitumorigenesis results in the present study, NSBB such as propranolol provides multifarious benefits and may be a crucial treatment in patients with AC.

In addition to NSBB, the simultaneous prescription for the comorbidities such as DM and cardiovascular diseases in AC may influence the incidence of HCC. For example, metformin has been reported to reduce the risk of HCC in patients with DM [35]; notably, it is also associated with tumor aggressiveness and drug resistance in advanced HCC [36, 37]. Besides, long-term aspirin use lowers the risk of HCC in the general population [38]; interestingly, lack of significant chemopreventive effect in cirrhotic patients has been proposed [39]. In general practice, the effects of these medications on HCC entail more studies to be unraveled.

As stated above, patients with cirrhosis carry a high risk of HCC. HCC screening in cirrhotic patients is an important issue in public health; the screening has been authenticated to decrease disease burden, reduce HCC rate, and improve survival conforming to standard practice [40–42]. In the view of the cost-effectiveness benefit, an annual incidence of HCC of 1.5% was regarded as the threshold of HCC screening [2]; although the annual incidence rates in AC are more than 1.5% in the majority of the literature, the result of 0.45% in a Danish national study puts the issue of HCC screening in AC patients in a dilemma [4, 43]. Hence, we managed to explore a more accurate incidence rate by means of a meta-analysis, and the data suggested that the current HCC screening policies could be applied to AC patients.

Even when we attempted to include as much research as possible into our meta-analysis, the majority of the published literature was carried out in Western countries; therefore, ethnical disparities might exist in our comparison. The only study undertaken in a Chinese population highlighted the superimposition of alcoholism on HBV-related HCC patients rather than on pure AC patients [17]. In addition, these studies did not analyze the incidence in terms of different severities, while the subjects in our study demanded hospitalization, and might result in more vulnerability to HCC compared to the general AC patients.

In spite of meticulous study design, our retrospective study has several limitations. First, the case number is comparatively few and thus may misestimate the accuracy of the incidence. In real-world practice, the adherence of medical instruction and the retention rate of observational study in patients with ALD are relatively low [44]; actually, concerning AC, the case number in our study is not less compared with the previous literature [23–25]. Second, although we applied PSM to reconcile the baseline bias, some information such as comorbidities was obtained retrospectively on the basis of medical records; therefore, we might have underestimated the proportion of the comorbidities. Also, the benchmark for the indirect comparison in the present study was the meta-analytic pooled annual incidence from the previous literature, among which part of the subjects might be treated with propranolol, which thus might offset the difference; however, even then, our data presented the effects of propranolol. Future works to elucidate the antitumorigenesis relationship and mechanism are warranted.

## 5. Conclusions

To conclude, we demonstrated the reduction of HCC incidence in patients with AC by propranolol; furthermore, we performed a meta-analysis of the annual incidence of HCC in AC. However, more epidemiologic data and larger-scale prospective cohort studies should be undertaken to ascertain the effects.

## Figures and Tables

**Table 1:** Baseline demographic data of subjects with alcoholic cirrhosis.

Variables	Patients with propranolol use, *N* = 23 (%)	Patients without propranolol use (number), *N* = 46 (%)	*P*
Male	20 (90.0)	43 (93.4)	0.743
Age^a^	52.5 ± 9.1	54.3 ± 12.0	0.858
Superimposed etiology			
HBV	6 (27.2)	17 (36.9)	0.367
HCV	1 (4.3)	6 (13.0)	0.365
Autoimmune hepatitis	0	2 (4.3)	0.31
Other CLD	0	0	N/A
Child-Pugh score^a^	7.5 ± 1.5	7.3 ± 1.5	0.575
Comorbidity			
IHD	1 (4.3)	0	0.31
Hypertension	5 (22.7)	11 (23.9)	0.547
COPD	1 (4.3)	1 (2.2)	0.365
DM	5 (22.7)	18 (39.1)	0.468
TB	0	1 (2.2)	0.484
Hyperlipidemia	0	0	N/A
ESRD	0	0	N/A
Cirrhosis duration (months)^a^	64.8 ± 17.3	63.8 ± 25.0	0.974
Incidence of HCC	1 (4.3)	11 (23.9)	0.043

CLD: chronic liver disease; COPD: chronic obstructive pulmonary disease; DM: diabetes mellitus; ESRD: end-stage renal disease; HBV: hepatitis B virus; HCV: hepatitis C virus; HCC: hepatocellular carcinoma; IHD: ischemic heart disease; N/A: not available; TB: tuberculosis. ^a^Mean ± standard deviation.

## Data Availability

The data used to support the findings of this study are available from the corresponding author upon request.
